# Thrap3 promotes R-loop resolution via interaction with methylated DDX5

**DOI:** 10.1038/s12276-021-00689-6

**Published:** 2021-10-25

**Authors:** Hyun Je Kang, Hye-jin Eom, Hongtae Kim, Kyungjae Myung, Hyug Moo Kwon, Jang Hyun Choi

**Affiliations:** 1 Department of Biological Sciences, Ulsan National Institute of Science and Technology (UNIST), Ulsan, 44919 Korea; 2 Center for Genomic Integrity (CGI), Institute for Basic Science (IBS), Department of Biological Sciences, Ulsan National Institute of Science and Technology (UNIST), Ulsan, 44919 Korea

**Keywords:** DNA damage and repair, Cancer genomics

## Abstract

Transcription-replication conflicts lead to DNA damage and genomic instability, which are closely related to human diseases. A major source of these conflicts is the formation of R-loops, which consist of an RNA-DNA hybrid and a displaced single-stranded DNA. Although these structures have been studied, many aspects of R-loop biology and R-loop-mediated genome instability remain unclear. Here, we demonstrate that thyroid hormone receptor-associated protein 3 (Thrap3) plays a critical role in regulating R-loop resolution. In cancer cells, Thrap3 interacts with DEAD-box helicase 5 (DDX5) and localizes to R-loops. Arginine-mediated methylation of DDX5 is required for its interaction with Thrap3, and the Thrap3-DDX5 axis induces the recruitment of 5’-3’ exoribonuclease 2 (XRN2) into R-loops. Loss of Thrap3 increases R-loop accumulation and DNA damage. These findings suggest that Thrap3 mediates resistance to cell death by preventing R-loop accumulation in cancer cells.

## Cancer: DNA damage associated with nucleic acid loops

A nuclear protein appears to inhibit cancer cell death by preventing the accumulation of nucleic acid structures called R-loops. R-loops are by-products of transcription, comprising two misaligned DNA strands and one RNA strand. They are involved in gene expression, but also threaten genome integrity and have been linked to the onset of neurodegeneration and cancers. A team led by Jang Hyun Choi and Hyug Moo Kwon, Ulsan National Institute of Science and Technology, South Korea, explored the role of Thrap3, a nuclear protein involved in RNA splicing, in R-loop-associated DNA damage. They found that Thrap3 binds to an enzyme essential for resolving R-loops. When the team suppressed Thrap3 expression, they saw an increase in R-loops in both normal and cancer cells. This R-loop accumulation significantly inhibited the growth of breast cancer cells.

## Introduction

R-loops are three-stranded nucleic acid structures consisting of a cotranscriptionally generated RNA-DNA hybrid and a displaced single-stranded DNA^1–4^. They have critical roles in a wide range of eukaryotic metabolic processes, such as chromosome segregation during mitosis, immunoglobulin class switching, DNA replication and repair, and transcription initiation/termination^3–5^. In addition, R-loops are involved in gene expression and rearrangement;^6^ however, they also threaten genome integrity, resulting in deleterious cellular effects. For example, abnormal accumulation of R-loops and subsequent collisions with replication forks is followed by DNA damage and genome instability^1,4,6,7^. R-loops have been postulated to be causative factors for many human diseases, such as neurodegenerative diseases, cancer, and autoimmune diseases^2^. Thus, proper regulation of R-loops in cells is essential.

DEAD-box helicase 5 (DDX5) is a critical factor in R-loop resolution; it associates with 5’-3’ exoribonuclease 2 (XRN2) and resolves R-loops at transcription sites^8,9^. DDX5 depletion leads to R-loop accumulation at these loci. Protein arginine methyltransferase 5 (PRMT5) binds to DDX5 and methylates it specifically at its RGG/RG motif^8,9^. This motif is required for the interaction of DDX5 with XRN2 and for the repression of cellular R-loop formation but is not essential for the enzymatic helicase activity of DDX5^8,9^. However, the factors involved in this mechanism of R-loop resolution are still not known.

In eukaryotic cells, pre-mRNA splicing and postsplicing events following transcription are essential for efficient gene expression^10^. Many proteins are involved in multiple stages of pre-mRNA processing, and these factors are components of the ribonucleoprotein complex called the spliceosome^10^. The nuclear protein Thrap3 (thyroid hormone receptor-associated protein 3) is an important component of the spliceosome in nuclear speckles and is a transcriptional coregulator^11^. Structurally, Thrap3 shares a homologous amino acid sequence with Bcl2-associated transcription factor 1 (BCLAF1) and contains arginine-serine rich domains, which are conserved features of the RNA splicing protein family^12,13^. Moreover, Thrap3 depletion causes cellular hypersensitivity to DNA-damaging agents^12^. Thus, Thrap3 is potentially involved in nuclear RNA transcription, processing, and metabolism, as well as in the maintenance of genomic stability.

In this study, we explored the role of Thrap3 in R-loop-associated DNA damage. We found that Thrap3 interacts with DDX5 and that this interaction is followed by XRN2 recruitment for R-loop resolution. Thrap3 specifically binds to arginine methylated-DDX5, suggesting that Thrap3 mediates resistance to DNA damage by preventing R-loop accumulation in oncogenic cells.

## Materials and methods

### Cell culture

U2OS cells, MCF7 cells, HEK-293T cells, and MEFs (mouse embryonic fibroblasts) were obtained from the American Type Culture Collection (ATCC, Manassas, VA, USA) and cultured in Dulbecco’s modified Eagle’s medium (DMEM) supplemented with 10% fetal bovine serum (FBS). To analyze the proliferation ability of MCF7 cells, 10^4^ cells were seeded in a 24-well plate, and the cell number was monitored using an IncuCyte FLR cell imaging microscope (Essen Bioscience, Ann Arbor, MI, USA) every day. Knockdown of Thrap3 in MCF7 cells was performed using lentiviral shRNA transduction. For lentivirus production, HEK-293T cells were transfected with lentiviral pLKO.1 shRNA plasmids (Sigma Aldrich, St. Louis, MO, USA) together with packaging and envelope plasmids. Culture media from transfected cells were collected and applied into MCF7 cells. Following infection, cells were selected with puromycin (VWR Chemicals, Radnor, PA, USA).

### Plasmids and siRNAs

The V5-tagged RNaseH1 (Cat# 111906) plasmid was purchased from Addgene (Watertown, MA, USA), and the Flag/Myc-tagged DDX5 plasmid was provided by the Center for Genomic Integrity (CGI) at UNIST. The FLAG/Myc-tagged Thrap3 plasmid was purchased from Origene (Rockville, MD, USA). Plasmids containing HA-tagged full-length and truncated Thrap3 were kindly provided by Woan-Yuh Tarn, National Taiwan University, Taipei, Taiwan. The sequences of the siRNAs were 5′-UUC UCC GAA CGU GUC ACG UTT-3′ for the control and 5′-GGU AUA AGC UCC GAG AUG A-3′ for human Thrap3. Transfection of the RNaseH1-V5, Flag/Myc-DDX5, and HA-Thrap3 plasmids and siRNAs was conducted with Lipofectamine 2000 and RNAiMAX transfection reagent (Invitrogen, Carlsbad, CA, USA), respectively. The transfection protocol was performed according to the manufacturer’s instructions.

### Cell lysis, western blotting, and immunoprecipitation

Whole-cell lysates were prepared by treating cold PBS-washed cells with RIPA lysis buffer mixed with 1% sodium deoxycholate, protease inhibitors (Roche, Basel, Switzerland), and phosphatase inhibitor cocktail (Sigma Aldrich, St. Louis, MO, USA). Collected protein extracts were quantified with a Pierce BCA Protein Assay Kit. Equal amounts of protein were loaded onto SDS–PAGE gels and transferred to a nitrocellulose membrane (GE Healthcare, Chicago, IL, USA). The membrane was blocked with 5% skim milk and incubated with each primary antibody overnight. Proteins were detected with chemiluminescent HRP substrate solutions (Advansta, Bering Drive San Jose, CA, USA). For immunoprecipitation, cleared cell extracts were incubated with specific antibodies overnight. Then, the protein-antibody samples were mixed with protein A/G agarose beads for 2 h and precipitated. Proteins were analyzed by blotting with antibodies specific for the following molecules: Thrap3 (F-10) (Santa Cruz Biotechnology, Cat# sc-133250), DNA-RNA Hybrid [S9.6] (Kerafast, Cat# ENH001), PCNA (PC10) (Santa Cruz Biotechnology, Cat# sc-56), DDX5 (A-5) (Santa Cruz Biotechnology, Cat# sc-166167), XRN2 (LifeSpan Bioscience, Cat# LS-B14272), mono- and dimethyl arginine (Abcam, Cat# ab412), β-Actin (Sigma–Aldrich, Cat# A5441), FLAG (Sigma–Aldrich, Cat# F7425), and HA-Peroxidase (3F10) (Roche, Cat# 12013819001).

### Immunofluorescence

U2OS cells were seeded on chamber slides. A day after seeding, cells were washed twice with PBS and permeabilized with 0.5% Triton X-100 for 5 min on ice before fixation with 4% paraformaldehyde for 15 min at RT. After washing with PBS, 0.5% Triton X-100 was added to permeabilize the cells. For chromatin-bound protein analysis, this series of steps was altered as follows: permeabilization with 0.5% Triton X-100 for 2 min and fixation with 100% methanol for 30 min. Then, cells were treated with blocking buffer for 1 h and incubated with primary antibodies overnight. After washing, cells were incubated with Alexa Fluor-conjugated secondary antibodies for 1 h. PBS-washed cells were visualized with a fluorescence microscopic imaging system. Antibodies against S9.6 (Kerafast, Cat # ENH001), Nucleolin (Cell Signaling Technologies, Cat# 14574), γH2AX (Millipore, Cat# 05–636), and 53BP1 (Cell Signaling Technologies, Cat# 4937) were used.

### Proximity ligation assay

The proximity ligation assay was carried out using a Duolink ≌ ® PLA fluorescence kit (Sigma Aldrich, St. Louis, MO, USA) with the standard protocols described as follows. Briefly, after the extraction, fixation, and permeabilization steps for slide-seeded U2OS cells, cells were blocked with 2% BSA in PBS for 1 h. Cells were incubated with primary antibodies and washed with PBS. Then, cells were incubated with premixed PLUS and MINUS PLA probes for 1 h. Samples were incubated with fluorescent ligation solution and amplification solution for 30 min and 100 min each, respectively. After a final wash, slides were mounted with DAPI and analyzed with a confocal microscope using a ×63 oil immersion objective. The numbers of nuclear PLA foci were quantified in the acquired images.

### DRIP (S9.6 DNA:RNA immunoprecipitation) assay

Cells were incubated with lysis buffer containing 85 mM KCl, 5 mM PIPES (pH 8.0), and 0.5% NP-40 for 10 min. Lysed cells were collected and centrifuged for 5 min at 3000 × *g*. The pellet (nuclei) was resuspended in RSB buffer (10 mM Tris-HCl (pH 7.5), 200 mM NaCl, and 2.5 mM MgCl_2_) with 0.2% sodium deoxycholate, 0.1% SDS, 0.5% Triton X-100, and protease inhibitor cocktail. Nuclei were sonicated for 10 min using a Diagenode Bioruptor. Then, the extracts were diluted in RSB buffer with 0.5% Triton X-100 and subjected to immunoprecipitation with the S9.6 antibody.

### EdU-incorporation assay for DNA replication

DNA replication was assessed by detecting EdU (5-ethynyl-2′-deoxyuridine) incorporation using a Click-iT™ EdU Cell Proliferation Kit (Invitrogen, Carlsbad, CA, USA) and Alexa Fluor™ 488 dye. Thrap3 knockdown and/or RNaseH1-overexpressing U2OS cells were labeled with EdU working solution for 15 min. After incubation, the medium was immediately removed, and the cells were fixed with 4% formaldehyde in PBS for 15 min and permeabilized with 0.5% Triton X-100 in PBS for 20 min. The permeabilization buffer was removed from the cells, and Click-iT™ reaction cocktail containing CuSO_4_ and Alexa Fluor azide solution in reaction buffer was added to the cells for 30 min. For nuclear visualization, DAPI was added. EdU-incorporation was detected with a fluorescence microscope, and the number of EdU-positive cells and the fluorescence intensity were analyzed.

### Purification and mass spectrometry analysis

HEK293 cells were transfected with the FLAG/Myc-tagged Thrap3 plasmid for ectopic expression of Thrap3, and lysates were incubated with anti-FLAG M2 agarose (Sigma Aldrich, St. Louis, MO, USA). After incubation, FLAG-tagged Thrap3 and interacting proteins were washed and eluted with 3X FLAG peptide (Sigma Aldrich, St. Louis, MO, USA). The eluted proteins were separated by SDS–PAGE, and the molecular characterization was analyzed through reversed-phase LC–MS/MS using an LTQ-Orbitrap high-resolution hybrid mass spectrometer (Thermo Fisher Scientific, Waltham, MA, USA).

### Statistical analysis

All data are presented as the mean ± standard error of the mean (SEM) values. The statistical significance of differences was assessed by Student’s *t* test or one-way analysis of variance (ANOVA). Statistical analyses were performed using Microsoft Excel or GraphPad Prism 9. All significance levels are expressed as *P < 0.05, **P < 0.01, ***P < 0.001, and ****P < 0.0001.

## Results

### Thrap3 colocalizes with R-loops, and Thrap3 depletion leads to R-loop accumulation

**Fig. 1:**
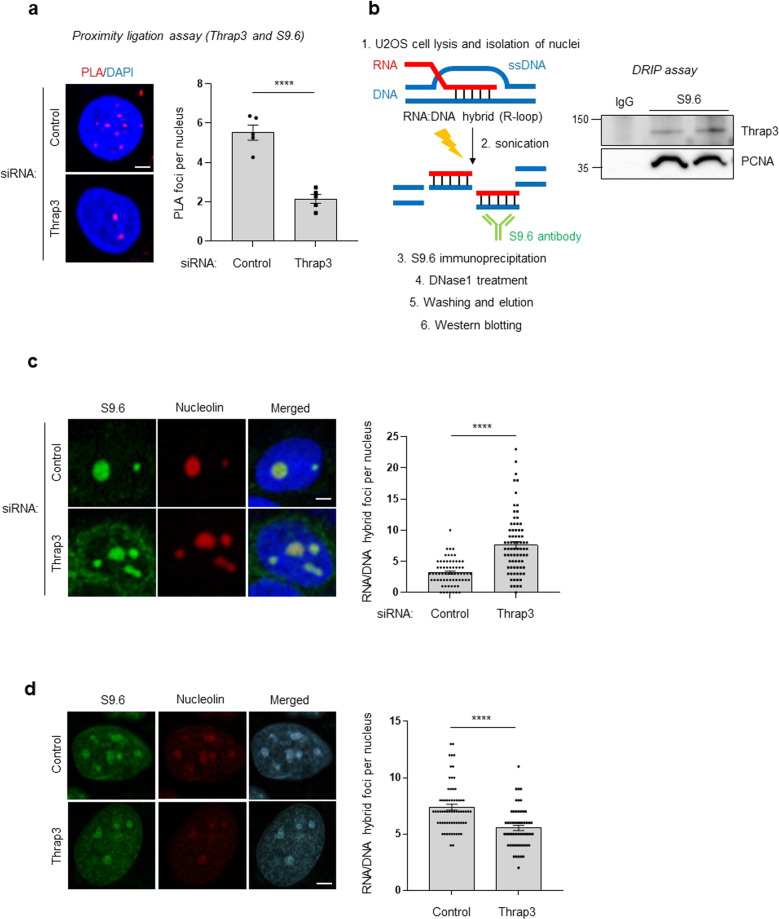
Thrap3 colocalizes with R-loops, and Thrap3 depletion leads to accumulation of R-loops. **a** U2OS cells were transfected with scrambled (control) or Thrap3-targeted (Thrap3) siRNA for 24 h. The cells were then subjected to a PLA to assess the physical closeness of Thrap3 and R-loops. (Left) representative images are shown; (right) percentages of PLA signal-positive nuclei were calculated from at least 30 cells. Mean ± SEM; *n* = 4; ****P < 0.0001. The scale bar represents 1 μm. **b** DRIP was performed, and the precipitates were analyzed by immunoblotting for Thrap3 and PCNA. (Left) Schematic of DNA-RNA hybrid immunoprecipitation (DRIP) assay. **c** U2OS cells were transfected with scrambled siRNA (control) or Thrap3-targeted (Thrap3) siRNA for 48 h. Cells were fixed and immunostained for S9.6 and nucleolin. (Top) representative images. (Bottom) the number of S9.6 foci per nucleus after subtraction of nucleolar signals was determined from at least 50 nuclei. Mean ± SEM, ****P < 0.0001. The scale bar represents 1 μm. **d** U2OS cells were transfected with empty vector (Control) and Thrap3 expression plasmids (Thrap3) for 48 h. Cells were fixed and immunostained for S9.6 and nucleolin. (Left) representative images. (Right) the number of S9.6 foci per nucleus was determined from at least 50 nuclei. Mean ± SEM, ****P < 0.0001. The scale bar represents 1 μm.

Thrap3 is an RNA-binding protein related to the DNA damage response through its involvement in RNA metabolism^11^. Therefore, we hypothesized that Thrap3 can associate with RNA in R-loop structures. Using a proximity ligation assay with the S9.6 antibody, which can detect R-loops, and an anti-Thrap3 antibody, we detected the colocalization of Thrap3 with R-loops in the nuclear region; in addition, depletion of Thrap3 using siRNA decreased the ligation signals in both cancer and normal (MEF) cells (Fig. 1a, Supplementary Fig. 1a, b). In addition, a DNA-RNA immunoprecipitation assay with the S9.6 and anti-Thrap3 antibodies confirmed the association between R-loops and Thrap3 in U2OS cells (Fig. 1b). Next, we investigated whether Thrap3 regulates the R-loop abundance. Immunofluorescence imaging showed that the abundance of R-loops was significantly increased when Thrap3 expression was knocked down by siRNA (Fig. 1c) but decreased when Thrap3 was overexpressed (Fig. 1d). Together, these findings suggest that Thrap3 interacts with and modulates R-loops.

### Thrap3 depletion inhibits DNA replication and induces double-strand break (DSB) formation

**Fig. 2:**
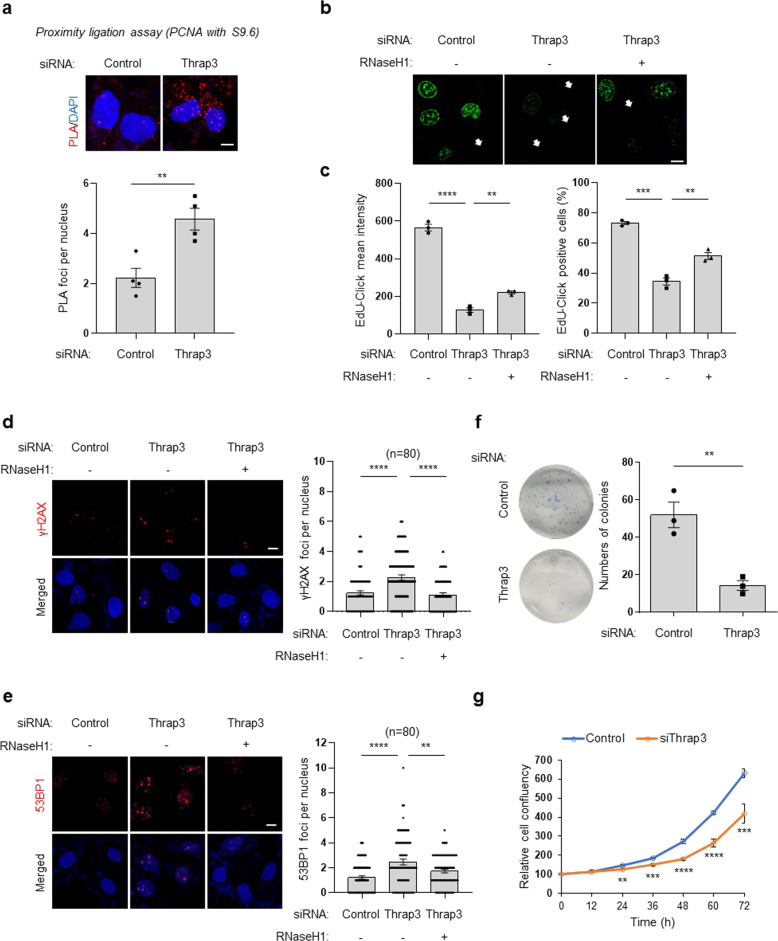
Thrap3 depletion inhibits replication and induces DSBs. **a** U2OS cells were transfected with scrambled (control) or Thrap3-targeted (Thrap3) siRNA for 24 h. The cells were then subjected to a PLA to assess the physical closeness of PCNA and R-loops. Representative images are presented. The scale bar represents 1 μm. **b**, **c** siRNA-transfected cells were transfected a second time with a plasmid expressing RNaseH1, as indicated. EdU-Click labeling was performed, and EdU incorporation was visualized. **b** Representative images. The white arrows indicate EdU-negative nuclei. **c** The Mean intensity and percentage of EdU-positive cells were measured from >30 cells. Mean ± SEM; *n* = 3; **P < 0.01, ***P < 0.001, ****P < 0.0001. The scale bar represents 2 μm. **d** siRNA-transfected cells were transfected a second time with a plasmid expressing RNaseH1, as indicated. Cells were fixed and immunostained for γH2AX. (Left) representative images. (Right) the number of γH2AX foci per nucleus after subtraction of nucleolar signals was determined from at least 30 nuclei. Mean ± SEM, ****P < 0.0001. The scale bar represents 2 μm. **e** siRNA-transfected cells were transfected a second tim**e** with a plasmid expressing RNaseH1, as indicated. The cells were fixed and immunostained for 53BP1. (Left) representative images. (Right) the number of 53BP1 foci per nucleus after subtraction of nucleolar signals was determined from at least 30 nuclei. Mean ± SEM; **P < 0.01, ****P < 0.0001. The scale bar represents 2 μm. **f** U2OS cells were transfected with scrambled (Control) or Thrap3-targeted (Thrap3) siRNA for 24 h. Cells were trypsinized, and 1000 cells were seeded in 6-well plates. The cells were allowed to proliferate for 12 days and were then fixed and stained with 10% methylene blue in 70% ethanol. The colonies were counted. (Left) representative images. (Right) the numbers of stained cell colonies per well. Mean ± SEM, **P < 0.01. **g** U2OS cells were transfected with scrambled (Control) or Thrap3-targeted (Thrap3) siRNA for 24 h. Cells were trypsinized, 10^4^ cells were plated in 24-well plates, and the cell confluence was monitored every 12 h using an IncuCyte FLR cell imaging microscope. Mean ± SEM; **P < 0.01, ***P < 0.001, ****P < 0.0001.

Depletion of Thrap3 increases R-loop accumulation. Because PCNA is abundant at the replication fork in transcription-replication collision (TRC) regions within R-loops^14,15^, we analyzed PCNA levels on R-loops when Thrap3 was depleted. PLA showed a significant increase in PCNA levels on R-loops (Fig. 2a). Because an unresolved R-loop causes DNA damage and promotes DNA strand breaks^1,6,7^, EdU incorporation in U2OS cells was measured under control and Thrap3 knockdown conditions to assess DNA replication. Decreased expression of Thrap3 inhibited DNA replication, and overexpression of RNaseH1, which is a ribonuclease responsible for R-loop resolution, restored the capacity for replication (Fig. 2b, c). Next, knockdown of Thrap3 was found to increase the numbers of γH2AX and 53BP1 foci, which are representative markers of DSBs, per nucleus (Fig. 2d, e). Overexpression of RNaseH1 reduced DNA double-strand breakage induced by Thrap3 depletion. These data suggest that Thrap3 protects against replication stress and DSBs by preventing R-loop accumulation. In addition, to determine whether Thrap3 depletion is associated with cell proliferation, we performed a colony formation assay (Fig. 2f) and IncuCyte cell imaging to monitor cell confluence (Fig. 2g). Depletion of Thrap3 resulted in inhibition of colony formation and cell proliferation. Thus, Thrap3 depletion inhibits DNA replication, resulting in retarded cell proliferation.

### Thrap3 binds to DDX5

**Fig. 3:**
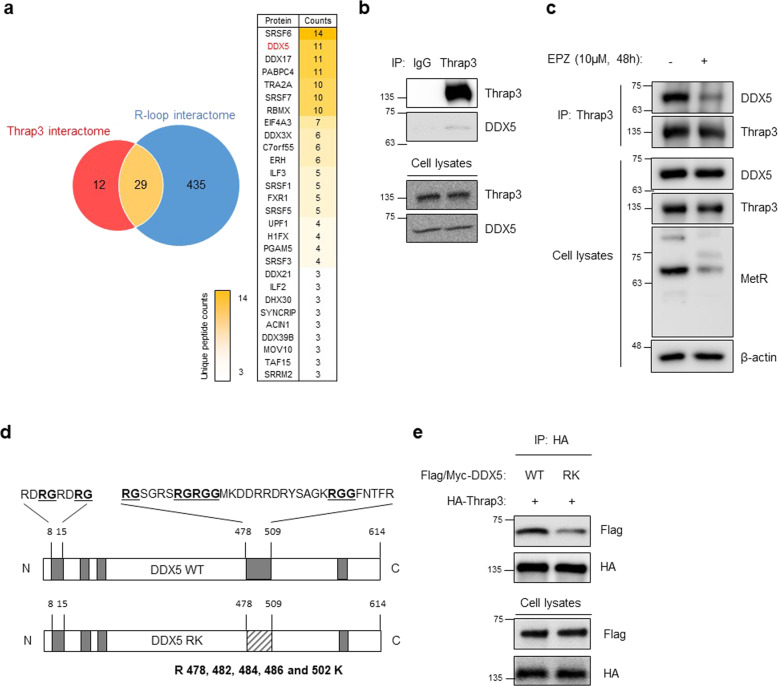
Thrap3 binds to DDX5 and XRN2. **a** Process for classifying Thrap3 binding proteins. **b** U2OS cell lysates were immunoprecipitated with normal IgG (IgG) or an anti-Thrap3 antibody (Thrap3). Precipitates and cell lysates were subjected to immunoblotting for Thrap3 and DDX5. **c** U2OS cells were treated with 10 μM EPZ015666 (EPZ) for 48 h. Cells were then immunoprecipitated with an anti-Thrap3 antibody (Thrap3). Precipitates and cell lysates were subjected to immunoblotting for DDX5, Thrap3 and MetR. **d** Schematic of DDX5 functional domains and motifs. **e** U2OS cells were transfected with Flag/Myc-tagged DDX5 and DDX5-RK combined with HA-tagged Thrap3. The cells were then immunoprecipitated with an anti-HA antibody (HA). Precipitates and cell lysates were subjected to immunoblotting for FLAG and HA.

Mass spectrometry was performed to identify Thrap3 interacting proteins and determine the mechanisms by which Thrap3 regulates R-loop levels and genomic stability. Proteins with significant expression changes of more than twofold upon modulation of Thrap3 expression were identified, and these proteins were then matched with the R-loop interactome dataset (PRIDE: PXD002960). The intersection of the two protein groups contained only 29 proteins. Among these 29 proteins, we focused on DDX5, which is known as an R-loop-regulating RNA helicase and a key Thrap3 interacting protein (Fig. 3a). We first examined the endogenous interaction between Thrap3 and DDX5 in U2OS cells. Lysed cells were incubated with the anti-Thrap3 antibody and control IgG, and DDX5 was coimmunoprecipitated with Thrap3 (Fig. 3b). DDX5 resolves R-loops and prevents the accumulation of R-loops by recruiting nucleases to methylated arginine-guanine motifs by PRMT5^8,9^. Therefore, we first checked whether Thrap3 regulates DDX5 methylation and found no difference in DDX5 methylation after Thrap3 depletion (Supplementary Fig. 2a, b). Therefore, it was also confirmed that the association between Thrap3 and DDX5 is altered by DDX5 methylation. U2OS cells were treated with 10 μM EPZ015666 for 48 h to inhibit PRMT5-mediated methylation. Inhibition of DDX5 arginine methylation also inhibited its protein interaction with Thrap3 (Fig. 3c). To further confirm the effect of EPZ015666, wild-type Flag/Myc-DDX5 and RK mutants were generated, in which five arginine sequences known to be methylated by PRMT5 were altered to lysine residues (Fig. 3d)^8,9^. The interaction between the DDX5 RK mutant and Thrap3 was diminished following EPZ015666 treatment (Fig. 3e). These results indicate that Thrap3 physically interacts with the RNA helicase DDX5.

### Thrap3 is associated with XRN2 on R-loops, and XRN2 is a downstream target of Thrap3

**Fig. 4:**
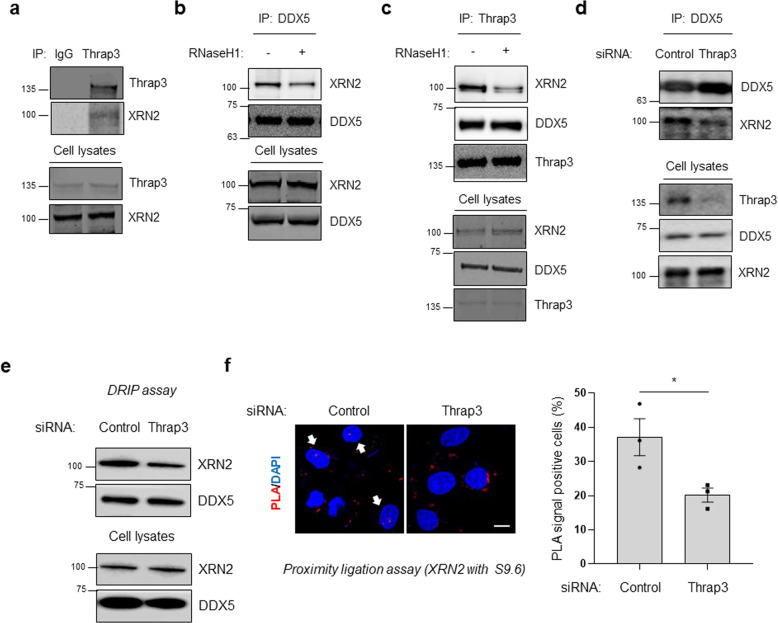
Thrap3 is associated with XRN2 on R-loops, and XRN2 is a downstream target of Thrap3. **a** U2OS cell lysates were immunoprecipitated with normal IgG (IgG) or an anti-Thrap3 antibody (Thrap3). Precipitates and cell lysates were subjected to immunoblotting for Thrap3 and XRN2. **b** U2OS cells were transfected with RNaseH1 overexpression plasmids for 24 h. Cell lysates were then immunoprecipitated with an anti-DDX5 antibody (DDX5). Precipitates and cell lysates were subjected to immunoblotting for XRN2 and DDX5. **c** U2OS cells were transfected with RNaseH1 overexpression plasmids for 24 h. Cell lysates were then immunoprecipitated with an anti-Thrap3 antibody (Thrap3). Precipitates and cell lysates were subjected to immunoblotting for XRN2, DDX5, and Thrap3. **d** U2OS cells were transfected with scrambled (control) or Thrap3-targeted (Thrap3) siRNA for 24 h. Cell lysates were then immunoprecipitated with an anti-DDX5 antibody (DDX5). Precipitates and cell lysates were subjected to immunoblotting for Thrap3, XRN2, and DDX5. **e** U2OS cells were transfected with scrambled (control) or Thrap3-targeted (Thrap3) siRNA for 24 h, and DRIP was then performed. Precipitates were analyzed by immunoblotting for XRN2 and DDX5. **f** U2OS cells were transfected with scrambled (control) or Thrap3-targeted (Thrap3) siRNA for 24 h. The cells were then subjected to a PLA to assess the physical closeness of R-loops and XRN2. (Left) Representative images are shown. (Right) Percentages of PLA signal-positive nuclei were calculated from at least 30 cells. Mean ± SEM, *n* = 3, *P < 0.05. The scale bar represents 1 μm.

XRN2 is involved in R-loop resolution via its exonuclease function and downstream proteins of methylated DDX5; therefore, it was determined whether Thrap3 also binds to XRN2. Immunoprecipitation data demonstrated that Thrap3 endogenously binds with XRN2 (Fig. 4a). To determine whether the interaction between DDX5 and XRN2 is R-loop-dependent, we tested the effect of RNaseH1 on this interaction. When RNaseH1 was overexpressed, protein binding between DDX5 and XRN2 was weakened (Fig. 4b). Interestingly, XRN2 recruitment to Thrap3 was also significantly decreased (Fig. 4c). Next, we examined whether Thrap3 affects the association between DDX5 and XRN2. Thrap3 siRNA- and control siRNA-transfected U2OS cells were lysed and immunoprecipitated with the anti-DDX5 antibody. The level of DDX5-interacting XRN2 was reduced under Thrap3 depletion conditions (Fig. 4d).

We assessed the interactions of Thrap3 with the R-loop resolution proteins DDX5 and XRN2 (Fig. 3a–e and Fig. 4a–d). However, the mechanisms by which R-loop resolution is mediated by Thrap3 are unknown. Given the result of each interaction between Thrap3, DDX5, and XRN2, we hypothesized that Thrap3 may mediate the recruitment of these R-loop resolution proteins. Therefore, a DRIP assay was applied under Thrap3 knockdown conditions, and Thrap3 depletion was found to cause a reduction in XRN2 binding on R-loops, although DDX5 binding was unchanged (Fig. 4e). In addition, Thrap3 knockdown cells showed significantly low PLA signals for binding between the S9.6 antibody and XRN2 (Fig. 4f). These results suggest that Thrap3-induced R-loop accumulation is partially caused by a decrease in XRN2 recruitment.

### Thrap3 is required for R-loop resolution in breast cancer

**Fig. 5:**
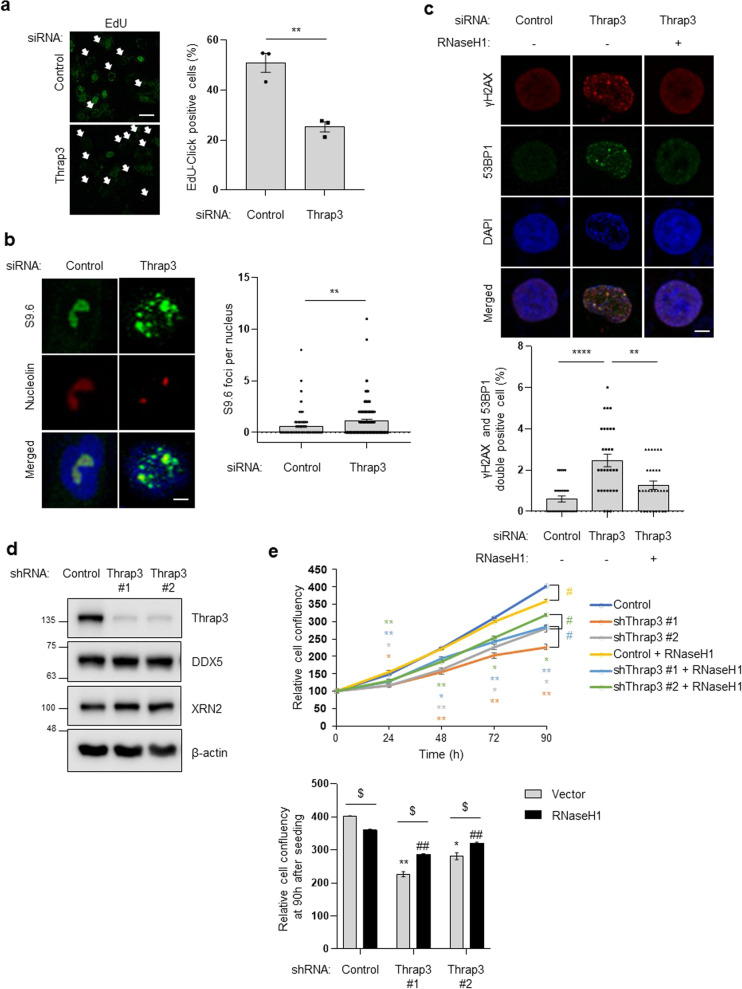
Thrap3 is required for R-loop resolution in breast cancer. **a** MCF-7 cells were transfected as indicated and were then subjected to EdU-Click labeling analysis. The scale bar represents 5 μm. (Left) representative images; the white arrows indicate EdU-negative nuclei; (right) the percentage of EdU-positive cells was measured from >30 cells. Mean ± SEM, *n* = 3, **P < 0.01. **b** Cells were transfected with scrambled siRNA (control) or Thrap3-targeted (Thrap3) siRNA for 48 h. Cells were fixed and immunostained for S9.6 and nucleolin. (Left) representative images; (right) the number of S9.6 foci per nucleus after subtraction of nucleolar signals was determined from at least 30 nuclei. Mean ± SEM, **P < 0.01. The scale bar represents 0.5 μm. **c** siRNA-transfected cells were transfected a second time with a plasmid expressing RNaseH1, as indicated. Cells were fixed and immunostained for γH2AX or 53BP1. (Top) representative images; (bottom) percentages of γH2AX- and 53BP1-positive nuclei were calculated from at least 30 cells. Mean ± SEM, *n* = 3, **P < 0.01, ****P < 0.0001. The scale bar represents 1 μm. **d** MCF-7 cells expressing scrambled (control) or Thrap3 (shThrap3) shRNA were analyzed by immunoblotting for Thrap3, DDX5, and XRN2. **e** MCF-7 cells expressing scrambled shRNA (control), Thrap3 shRNA #1 (shThrap3 #1), or Thrap3 shRNA #2 (shThrap3 #2) were transfected with RNaseH1 overexpression plasmids. Cells were then analyzed using an IncuCyte assay to confirm cell growth. (Top) the relative cell confluence was measured daily for a 90 h period. Mean ± SEM; *n* = 3; *P < 0.05, **P < 0.01. ^#^*P* < 0.05, comparison between vector- and RNaseH1-transfected cells. (Bottom) Representative cell confluency at 90 h after seeding. Mean ± SEM; n = 3; *P < 0.05, **P < 0.01, ^##^*P* < 0.01, comparison to control cells. ^$^*P* < 0.05, comparison between vector- and RNaseH1-transfected cells.

Next, the role of Thrap3 in R-loop resolution under pathophysiological conditions was investigated. As observed in U2OS cells, Thrap3 depletion reduced EdU-incorporation in MCF7 breast adenocarcinoma cells, indicating decreased DNA replication (Fig. 5a). In addition, Thrap3 knockdown in MCF7 cells induced R-loop accumulation in nuclei (Fig. 5b). Thrap3-mediated R-loop accumulation induced γH2AX and 53BP1 localization in the nucleus, and resolution of R-loops through overexpression of RNaseH1 alleviated DNA double-strand breakage caused by Thrap3 knockdown (Fig. 5c). The growth of Thrap3 knockdown MCF7 breast cancer cell lines was significantly inhibited, and RNaseH1 expression restored the cell proliferation capacity (Fig. 5d, e). These data indicate that Thrap3 is required for R-loop resolution in MCF7 cells.

## Discussion

**Fig. 6:**
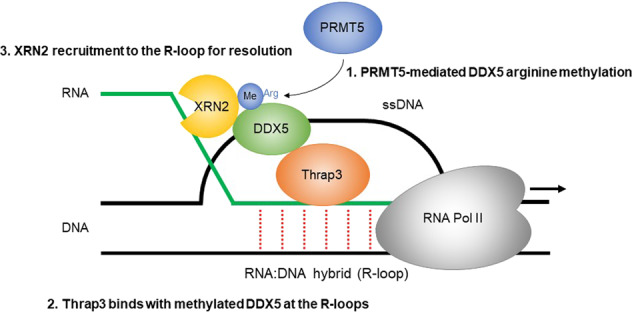
Model for the function of Thrap3 in R-loop resolution. Working hypothesis of the function of Thrap3 in R-loop resolution. The RNA-binding protein Thrap3 localizes at R-loops. In addition, PRMT5 induces methylation of RG/RGG motifs in DDX5 at R-loops. Thrap3 binds to methylated DDX5, and this binding is followed by XRN2 recruitment. XRN2 resolves R-loops through its exonuclease activity.

R-loops have important physiological roles and have been implicated in the pathogenesis of various diseases^1,2,16^. Although the factors and cellular processes associated with R-loops have been extensively studied, many aspects of R-loop biology and R-loop-mediated genome instability remain unclear. This study focused on the function of Thrap3 in R-loop-associated DNA damage in cancer cells and identified Thrap3 as a novel regulator of R-loop-associated DNA damage. Thrap3 is recruited to R-loop-forming loci, and loss of Thrap3 increases R-loop accumulation and DNA damage, thereby increasing the amount of replication stress, demonstrating that Thrap3 plays an important role in resolving R-loop-associated DNA damage in cancer cells. Thrap3 is also required for the recruitment of XRN2 into R-loops for their resolution. Similar results were obtained by knocking down PRMT5 or inhibiting DDX5 expression^8^. These findings provide new insights into the molecular mechanisms underlying cellular DNA damage and the repair response, as well as the novel functions of Thrap3 in R-loop biology (Fig. 6).

RNA processing factors play various roles in the DNA damage response^17^. They regulate mRNAs encoding DNA repair factors or are involved in DNA repair by associating with DNA damage response factors^17^. However, the mechanism of RNA processing factor-dependent R-loop resolution remains unknown. Interestingly, it has been reported that Thrap3 and DDX5 have common binding proteins related to RNA processing factors, including SRSF1 and TAF15^18^. Loss of SRSF1, a well-known splicing factor, induces R-loop accumulation and genomic instability^6,19^. In addition, TAF15 is recruited to DNA damage sites with other R-loop suppressor proteins, including EWSF1, SAFA, and FUS, and prevents R-loop accumulation^20–22^. Thus, there is a possibility that Thrap3 has various downstream targets for R-loop removal via its interaction with DDX5. However, more precise mechanistic studies on Thrap3-mediated R-loop resolution are needed. In addition, Thrap3 has another binding candidate, SRSF6 (list of top-ranked Thrap3 binding proteins, Fig. 3a). SRSF6 regulates constitutive splicing and modulates alternative splice site selection^23^. Although SRSF6 has not been characterized as a DNA damage- or R-loop-mediated factor, Thrap3 might function in genomic instability with SRSF6 through a physical interaction.

Proteins that regulate gene transcription also have functions in other DNA processes, such as DNA replication and repair^24–29^. For example, RNA m6A (methylation of an A base), which is important for many RNA metabolic processes, is highly enriched at sites of UV irradiation-induced DNA damage for DNA polymerase kappa (POLK) recruitment^30^. In addition, TonEBP, a pleiotropic transcription factor, plays a role in the DNA damage response through interaction with various proteins related to PCNA polyubiquitination or m6A RNA methylation^27,28^. According to our unpublished data, Thrap3 binds to TonEBP and induces PCNA ubiquitination under conditions of DNA damage. Consistent with this idea and our preliminary data, the RNA processing factor Thrap3 might participate in the DNA damage response by regulating RNA modification in cooperation with various types of binding proteins (TonEBP, RNaseH1, PCNA, etc.).

DDX5 has been studied for its function in cellular RNA processing^31^. Its role in transcription, as suggested by studies in *Drosophila* and yeast DDX5 homologs, is to activate RNA release from chromatin^32,33^. DDX5 also plays a role in transcription initiation through the resolution of R-loop enrichment in promoters and transcription start site (TSS) regions with a high GC content^34^. However, how DDX5 is recruited to these sites is not known. Our data suggest that Thrap3 is associated with DDX5 for R-loop resolution and that overexpression of RNaseH1 restored the effect of Thrap3 depletion on cell proliferation; therefore, it is possible that Thrap3 regulates DDX5 recruitment to promoter and/or TSS regions and DNA damage lesions. It has been reported that treatment of human breast cancer cells with 17β-estradiol (E2) dramatically increases the accumulation of R-loops, particularly in regions of the genome containing estrogen-induced genes^35^. Interestingly, estrogen receptor α (ERα) specifically interacts with Thrap3 under conditions of E2 exposure^36^. Because E2-dependent R-loop generation and rearrangement in breast cancer cells are mainly enriched at E2-responsive genomic loci, it is possible that Thrap3 might be localized to E2-responsive genomic loci with ERα and that this localization is followed by DDX5 recruitment for precise removal of cotranscriptional R-loops in breast cancer cells, which is critical for cancer progression.

## Supplementary information

Supplementary Information
