# Supplementary material for: Thrap3 promotes R-loop resolution via interaction with methylated DDX5
Source: Exp Mol Med. 2021 Oct 25;53(10):1602–11. doi: 10.1038/s12276-021-00689-6 (PMC8569202; doi:10.1038/s12276-021-00689-6)
Supplement: Supplementary file 1 — Supplementary Information [file 12276_2021_689_MOESM1_ESM.pdf]

Thrap3 promotes R-loop resolution via interaction with methylated DDX5

Hyun Je Kang[1,†], Hye-jin Eom[1,†], Hongtae Kim[1], Kyungjae Myung[1,2], Hyug Moo Kwon[1,*], and Jang Hyun Choi[1,*]

Supplementary Information

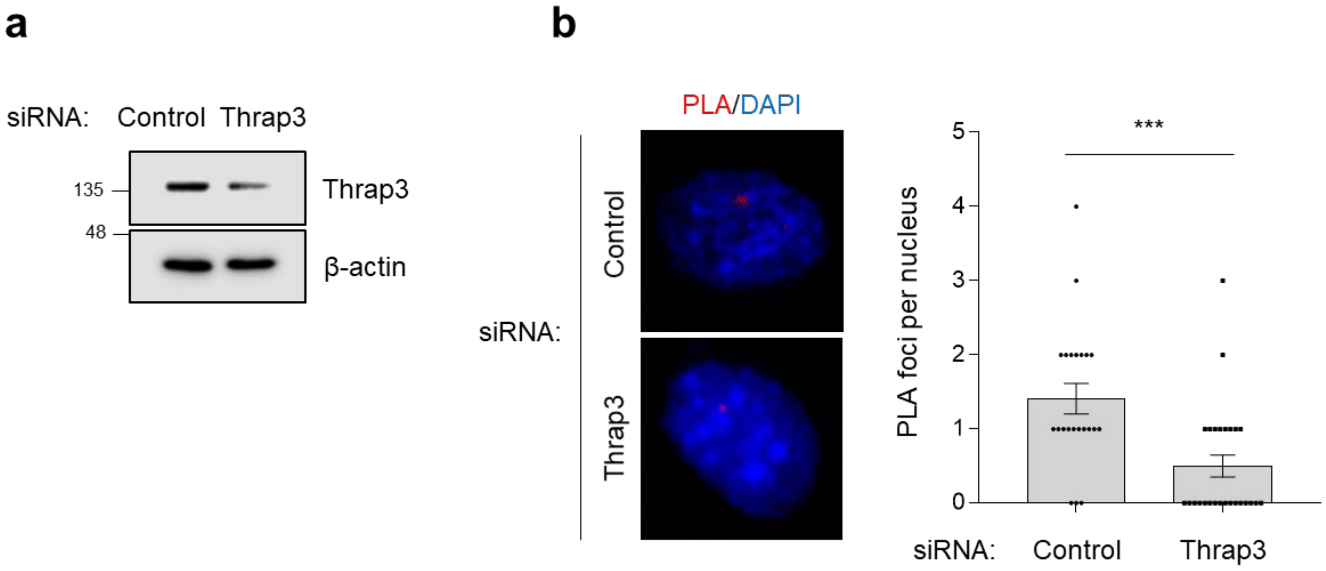

Supplementary Figure 1. Thrap3 co-localizes with R-loops and Thrap3 depletion leads to accumulation of the R-loops in MEFs: a) MEF cells were transfected with scrambled (control) or Thrap3-targeted siRNA (Thrap3) for 48 h. Protein lysates were extracted and Thrap3 protein expression was confirmed by western blot. β-actin was used as normalizing protein. **b)** MEF cells were transfected with scrambled (control) or Thrap3-targeted siRNA (Thrap3) for 48 h. The cells were then subjected to PLA assay between Thrap3 and S9.6. (left) representative images are shown; (right) PLA signal-positive nuclei were counted from at least 20 nuclei.

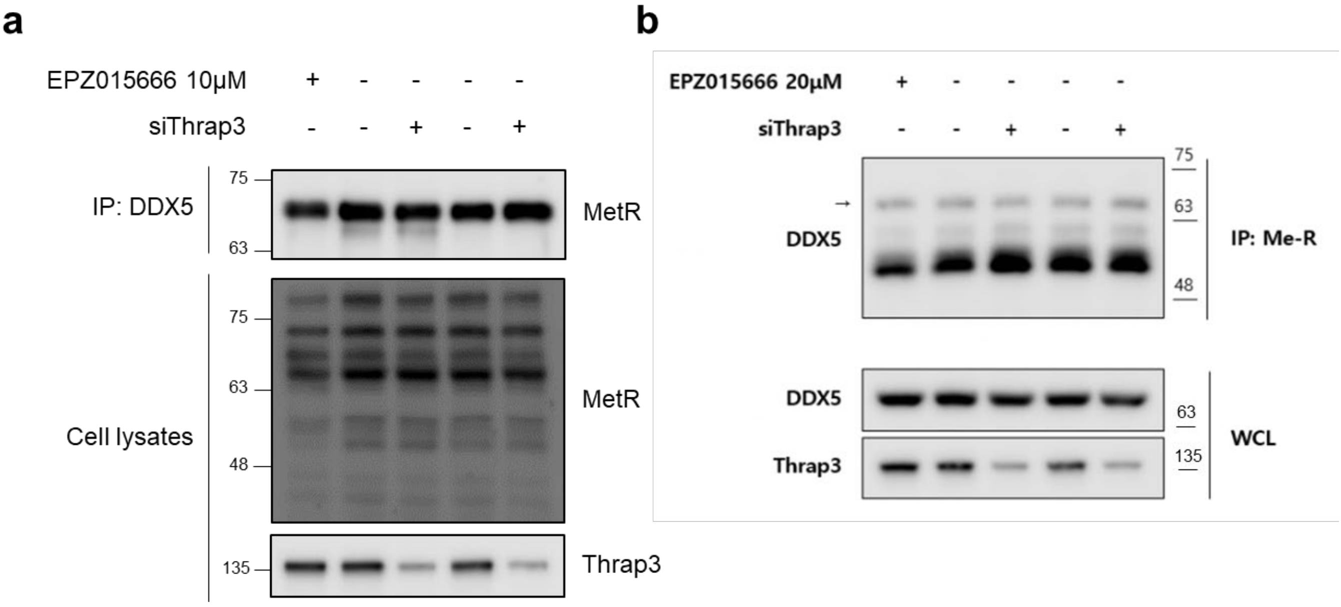

Supplementary Figure 2. Thrap3 depletion did not affect methylation of DDX5: a) U2OS cells were treated with 10 μM of EPZ015666 or transfected with siRNA targeting Thrap3, as indicated in figure. 48 h after treatment, cells were lysed and immunoprecipiated with DDX5 antibody. Precipitates and cell lysates were blotted for MetR and Thrap3. **b)** U2OS cells were treated with 20 μM of EPZ015666 or transfected with siRNA targeting Thrap3. 48 h after treatment, cells were lysed and immunoprecipiated with MetR antibody. Precipitates and cell lysates were blotted for DDX5 and Thrap3.
